# Breakdown of Methods for Phasing and Imputation in the Presence of Double Genotype Sharing

**DOI:** 10.1371/journal.pone.0060354

**Published:** 2013-03-28

**Authors:** Carl Nettelblad

**Affiliations:** 1 Division of Scientific Computing, Department of Information Technology, Uppsala University, Uppsala, Sweden; 2 Laboratory of Molecular Biophysics, Department of Cell and Molecular Biology, Uppsala University, Uppsala, Sweden; University Medical Center Utrecht, The Netherlands

## Abstract

In genome-wide association studies, results have been improved through imputation of a denser marker set based on reference haplotypes and phasing of the genotype data. To better handle very large sets of reference haplotypes, pre-phasing with only study individuals has been suggested. We present a possible problem which is aggravated when pre-phasing strategies are used, and suggest a modification avoiding the resulting issues with application to the MaCH tool, although the underlying problem is not specific to that tool.

We evaluate the effectiveness of our remedy to a subset of Hapmap data, comparing the original version of MaCH and our modified approach. Improvements are demonstrated on the original data (phase switch error rate decreasing by 10%), but the differences are more pronounced in cases where the data is augmented to represent the presence of closely related individuals, especially when siblings are present (30% reduction in switch error rate in the presence of children, 47% reduction in the presence of siblings).

The main conclusion of this investigation is that existing statistical methods for phasing and imputation of unrelated individuals might give results of sub-par quality if a subset of study individuals nonetheless are related. As the populations collected for general genome-wide association studies grow in size, including relatives might become more common. If a general GWAS framework for unrelated individuals would be employed on datasets with some related individuals, such as including familial data or material from domesticated animals, caution should also be taken regarding the quality of haplotypes.

Our modification to MaCH is available on request and straightforward to implement. We hope that this mode, if found to be of use, could be integrated as an option in future standard distributions of MaCH.

## Introduction

Genome-wide association studies (GWAS) have shown great success in unravelling the genetic variation underlying many important traits and disease complexes in natural human populations [1], [2]. Imputation of marker data has been suggested, both as a way to augment missing or sparse genotype data based on reference haplotypes from sequenced reference haplotypes [3], and in order to reconcile study cohorts assembled from genotyping efforts using different SNP panels [4]. The process of imputation consists of inferring the genotype phase for all markers, and then finding the best corresponding genotypes in the reference population, for those markers that are missing in experimental data. The underlying assumption is that short haplotype blocks are most likely preserved over the course of many generations. Thus, a suitable panel of reference haplotypes can be highly informative for genotypes not observed directly, and increase detection power.

Panel sizes are constantly growing, from the tens or hundreds in original Hapmap populations [5], into currently 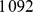 high-quality human genomes from the 1000 Genomes Project [6], [7]. However, some popular algorithms for genotype imputation scale as 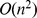 [8], [9] in runtime per study individual with unknown phases, where 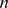 is the total number of haplotypes (haploid references and study). An increase in panel size by a factor of 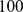 might therefore increase runtime by a factor of 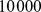, exhausting computational resources. Other approaches exist [10], but reduce computational complexity by making additional approximations.

Due to the rapid increase in the computational complexity of Markov model phasing with increasing reference population size, it has been suggested to infer the phases using only the study population (or a subset thereof), followed by imputing genotypes into this fixed (pre-phased) haplotype set [11]. This operation reduces the computational complexity, allowing much larger reference panel sizes. However, as no known fixed haplotypes are available during pre-phasing, the Markov chain approaches used in the most popular pre-phasing schemes become more sensitive to the problem of chain trajectories getting stuck in local minima. In this paper we describe a specific scenario causing the model optimization to stall. We show the extent of the problem with experimental data, and suggest a possible modification of the MaCH [8] algorithm successfully circumventing the issues.

## Materials and Methods

Most hidden Markov model approaches for phasing of genotype data lacking a pedigree share several characteristics [12]. A state in the model consists of a haplotype pair, meaning that an observed unordered genotype pair in one individual corresponds to a pair of haplotypes from other individuals. With a proper selection of transition probabilities, blocks of the genome will be attributed to identical states, reflecting identical ancestry. The posterior probabilities for the state distribution can be found at each position, and putative haplotype candidates can be determined by sampling from that distribution. By iterating over all individuals, the undetermined (sampled) haplotypes can be successively improved.

**Figure 1:**
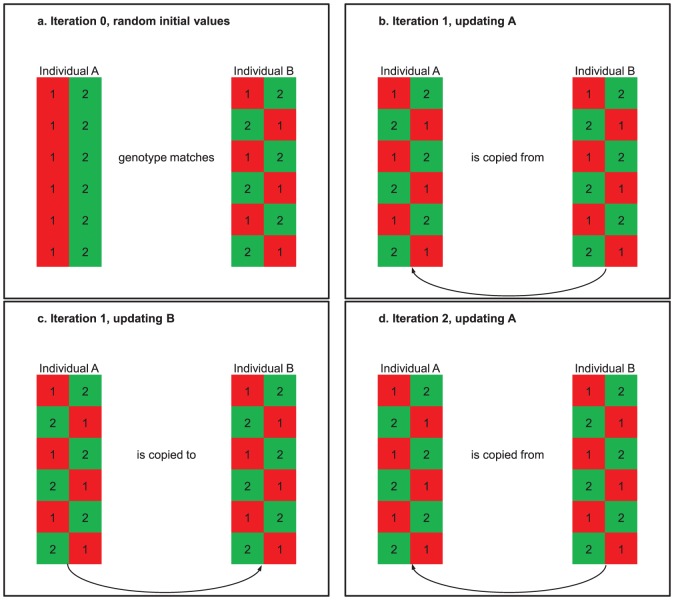
Bad MCMC mixing for cases of double genotype sharing. MaCH and similar approaches implement a Markov-chain Monte Carlo scheme where in each iteration the individual genotype resolutions are updated one by one, by mapping the genotypes. If two individuals contain identical marker genotypes for a longer stretch of markers, the Hidden Markov Model will give the other individual a probability approaching 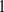. When no reference haplotypes are provided, all haplotype data is initialized randomly. In this series of panels, individuals 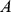 and 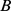 are initialized differently (a). In panel (b), A is updated. With high probability, the existing (random) haplotype resolution from 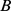 is copied. When 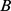 is updated (c), 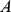 is sampled with high probability, replicating the original random data for 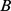. In iteration 2, 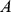 is updated again (d), but again 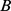 is sampled with high probability. Since any haplotype resolution for 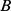 will match the genotypes for 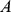, there is no pressure to identify a better resolution. The two individuals form a local feedback loop with no true mixing in the Markov chain. Our modified algorithm lowers the probability of sampling from a mirror individual (like the pair of 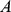 and 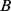), thus allowing haplotypes from other individuals in the dataset to influence the final resolution. Similar cases can also arise with larger groups of individuals than 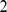. Those are handled successfully by our remedy, as well.

Consider that such a successive improvement is underway, and that the next step is to sample new haplotypes from the posterior distribution for individual 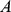. This step is shared by e.g. MaCH and IMPUTE2. Also assume that individuals 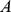 and 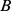 are completely identical, over a major stretch of a chromosome. In this case, a problem arises. This is not an uncommon case, rather, it is sufficient that the individuals are ordinary full siblings for this to occur. Approximately 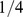 of the total genome for a pair of siblings will consist of such very long regions, as crossover events are relatively far apart relative to the marker density in modern maps. The posterior probability when individual 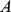 is analyzed will be completely dominated by the haplotypes for 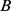 in such a region. However, this dominating effect will only be justified if the haplotypes for 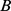 are truly correct. Since genotypes match in every position, *any* haplotype resolution for 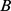 will have a dominating influence on 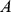. Correspondingly, any haplotype resolution for 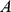 will have a dominating influence on 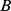. In an iterative optimization scheme starting out from randomly initialized haplotypes without an external reference, the pair of 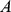 and 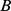 will be locked in a local minimum very close to the starting point. This structure is illustrated in Figure 1.

If transition probabilities are also iteratively updated based on observed data, the problem is further compounded. The single very favorable state also makes transition events rare. Transitions then become even more infrequent in later iterations, further decreasing the probability of sampling another haplotype.

The effect is not necessarily confined to two individuals. If a larger set of individuals share a comparatively long region in both chromatids, i.e. carry identical-by-state genotypes for all markers in a long region, the same kind of lock-in effect will appear. The state distribution will consist of a mix of states, but it will be almost totally occupied by different combinations of haplotypes from the set of similar individuals, and the sampling at each marker in each iteration will almost always be drawn from this set, thus only reflecting the initial randomization of phase.

Our proposed remedy to this is to keep the current model formulation, but improving the mixing properties of the sampling process. The sampling process in MaCH [8] starts from the last marker, iteratively going backwards, sampling based on the forward probabilities given the state at the previous marker sampled. Specifically, there is a vector for all unique pairs of 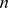 haplotypes. What should be filtered out is those cases where the pair taking one haplotype from 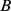 and one from 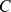 (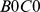) is just as likely as taking the other haplotype from both individuals (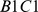). When that is the case, any haplotype resolution would match, as per the reasoning above. Thus, the match can be uninformative, causing a local (incorrect) minimum to be maintained. The (non-normalized) sampling probability used for 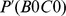 is, with our modification, instead 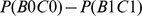 (assuming the result is positive, otherwise capped to a small 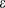), where 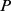 is the forward probability. In the case where 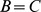, the result is that sampling the “copy another individual” pair 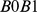 is precluded, as 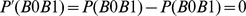. By only modifying the sampling probability, our approach does not affect the overall structure of the model. The probability distribution is re-normalized after the subtraction step outlined above.

### Experiments using Hapmap population data

In order to verify the extent of the problem when phasing a small set of realistic dense human data, we used the 60 first chromosome 21 haplotypes (30 parents with 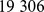 markers) of the phased Hapmap3 release 2 Utah residents with Northern and Western European ancestry from the CEPH collection (CEU) trios [13]. The full set of identified SNPs available in the phased data was used. Every second marker was cleared to be used as a test set for imputation. The individuals were not supposed to be closely related since the data only consisted of the parental generation.

In order to introduce a high degree of double genotype sharing, which is the problem condition we are interested in, we also created modified datasets based on this original data. These included adding back the child in each trio set (45 individuals in total); and two sets of monozygotic twins to the parents, one creating an overall double haplotype sharing consistent with the presence of full siblings (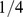, twins for the 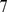 first parents, 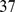 individuals in total), and one introducing a monozygotic twin to each parent (60 individuals in total). Using full twins along the whole genome should be similar to resampling true full siblings, since typical regions shared in this manner with this level of relatedness should be of chromosome-like length (tens of cMs/Mbps).

Phasing in MaCH was run for 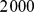 iterations. While benefits from more than 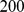 iterations were limited, we were interested in discovering whether the near-asymptotic behavior of the original MaCH and our modified version were identical. It could be argued that the improved mixing of our modification would only speed up convergence, but not affect the results after a high number of iterations.

After phasing, the number of switch errors in the phase sequences were counted compared to the original phased data. The switches (as defined in [14]), or flips, were only counted for the 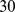 original parents for all datasets, in order to make the numbers directly comparable. The phased data as well as estimated genotype error and recombination rates were then fed to minimac for imputation using the remaining 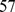 parents in the trio dataset as a reference panel.

## Results

We have implemented the modification outlined in the Methods section in MaCH. The change could easily be added to the main source tree as an extra option. Instructions on how to make the corresponding changes to the source are available on request. The performance of our modified approach is demonstrated in Table 1, with comparisons relative to an unmodified version of MaCH 1.0.17. Clear improvements are demonstrated for the number of switches needed to represent the true haplotypes (as reported by the Hapmap consortium), as well as in imputation accuracy, even for a dataset consisting of supposedly unrelated individuals. When artificial siblings were added, compounding the problem, the effects are far more drastic. Our modified version results in modest improvements in switch error rate as well as imputed alleles for the unmodified dataset, despite the fact that no long regions of double genotype sharing would generally be expected in unrelated individuals.

**Table 1:** Comparison between original and modified MaCH.

	Original MaCH		Modified MaCH	
Dataset	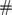 switches	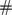 incorrect imputed alleles	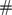 switches	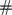 incorrect imputed alleles
Trio parents (no children)	5408	3730	4915	3566
Trio parents and children	1907	3261	1350	3217
Full siblings to parents	9657	4611	5096	3616
Monozygotic twins to parents *	42074	8787	6309	4016

Comparison between original MaCH and a modified version with our remedy, showing both the total number of switch errors and the number of incorrectly imputed alleles. The comparison is based on the 30 first phased Hapmap3 release 2 CEU trio parents [13]. Four versions are used: 1. the original dataset (only parents), 2. including their children, as well as 3. simulating siblings to parents, 4. simulating twins to parents. When children are excluded and no virtual siblings are present, no known relationships exist between the individuals in the dataset. Imputation performance was verified by reconstructing the half of the marker set (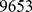) that was left out, using minimac [11], employing the remainder of the phased CEU trio data (57 individuals) as reference panel. All MaCH runs were executed for 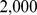 iterations, with 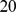 rounds for minimac. Metrics are reported for only the 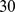 original individuals, in order to aid comparisons.

* In this case, the minimac run starting from the recombination frequencies determined by the original MaCH failed to converge at all, with errors for all markers. The results for original MaCH in this table row are based on the pre-phased haplotypes from original MaCH, but starting out with the recombination frequencies from the modified version, in order to allow the minimac imputation to complete at all.

These results led to an investigation of the amount of double genotype sharing in additional detail. The average best match for any parent individual to some other parent individual was slightly above 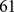, i.e. if a marker in this dataset is chosen at random for some individual, it will on average be part of a stretch with double genotype sharing of total length 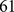. The longest matching region between any pair of individuals was 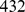 markers in length, corresponding roughly to 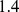 cM for the marker density in the Hapmap data used, indicating that the individuals are indeed not extremely closely related. To perform haplotype inference at all with these algorithms, single haplotype sharing must be present between individuals. This implies single genotype sharing as well. The average for single genotype sharing in this dataset is a region 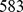 markers in length, with a maximum of 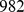 markers. The probability of a single chromatid being shared identical by descent in some region is naturally higher than the same condition holding for both chromatids at the same loci.

For the other cases, where double genotype sharing was explicitly introduced, the differences detected between the methods are drastic. The switch error rate at most rises by 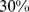 for our modified version (in the case of simulated MZ twins). The original MaCH phasing breaks down in this scenario, with an almost eight-fold increase in the switch error rate, and a doubling of imputation errors. In the more plausible scenario of siblings rather than twins being present, the original MaCH error rate still increases by over 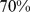.

**Figure 2:**
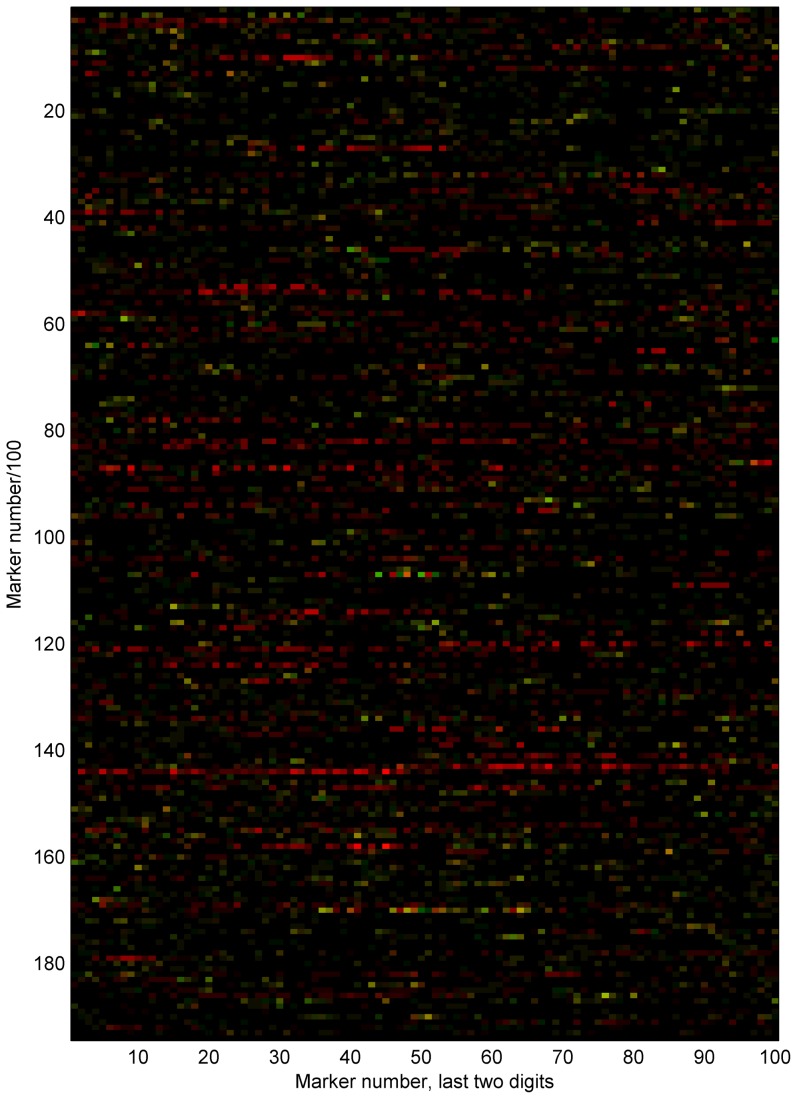
Comparison of switch error locations. Switch errors for all markers for CEU trio parents on chromosome 21 plotted in order left-right, top-down (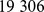 markers). For each marker, red color intensity indicates the switch error rate for all 30 parents using the original MaCH 1.0.17 algorithm, while green intensity indicates the error rate using our proposed modification. Hence, yellow color indicates regions where errors are shared. The issue of bad chain mixing we describe for the original algorithm manifests as contiguous (horizontal) blocks of repeated switch errors using the original approach, while the error rate using the modified algorithm is 50% lower in total. The errors in the modified algorithm consist of events more evenly distributed. Several of those error locations coincide with errors from the original method. This figure also shows that even if overall haplotype quality in terms of error rate would be acceptable, some regions can still be heavily affected, and paradoxically those regions are the ones where multiple individuals share both haplotypes identical by descent.

Although the differences in results are modest in some cases, we have observed the original MaCH method to be much more sensitive to details in input data structure. We tested including all markers, ignoring the step of leaving every second out for imputation purposes. This increased the switch error rate dramatically for the original MaCH, but only resulted in a modest increase in our modified version. The total number of switch errors for the 30 CEU parents tested in our experiments, when no markers are masked, are 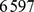 for our modified version and 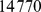 for the original MaCH. Figure 2 shows the switch error rate (out of the total of 30 parents) for the unmasked dataset, indicating that the original MaCH version will sometimes create long regions of repeated phasing errors that also coincide between multiple individuals, as predicted.

## Discussion

We think that our results regarding the extent of deterioration in haplotype quality when some types of related individuals are included in the data should be of interest to all situations where imputation or phasing based on Markov model methods are used, but especially so in the case where pre-phasing is performed followed by imputation with e.g. minimac, or when it is known that some of the individuals to be phased might be closely related. It is also relevant to point out that even in a dataset with supposedly unrelated human individuals, our remedied version reduces the switch error rate by 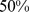 when no markers were masked.

It should be noted that the degree of relationship required for the issue of double genotype sharing to be present does not have to be as close as full siblings. Rather, the relevant condition is whether there is some probability that two individuals share both homologues of a certain region identical by descent. This could be the case for e.g. double cousins, but the condition could also hold for far shorter regions (but still on the range of multiple Mbps) in relatively isolated populations with little historical exchange of genetic material. The issue described could be even more serious for analyses in non-human species, where no reference haplotypes at all might exist, or where double genotype sharing might be aggravated due to (artificial or natural) inbreeding patterns.

Indeed, relatedness to the level of causing extensive double genotype sharing in some regions between some individuals could be considered a necessary condition for this type of phasing algorithms to work at all, since they rely on regions of *single* haplotype sharing between individuals being present in order to infer the correct haplotypes. The expected regions of *double* genotype/haplotype sharing will be much shorter, but the presence of some such regions between some individuals will still be expected with data showing enough haplotype sharing to allow successful inference.

The effects seen in switch error rate are not fully reflected in the imputation error rate. We suggest that this is due to the insufficient size of the very limited reference panel used in this specific experiment. The quality of the pre-phasing only influences imputation quality when the reference set contains matches to the true haplotypes.

If one is reluctant to use our remedy or other modifications of existing haplotype inference approaches, we still suggest investigating the quality of phasing, both in pre-phasing schemes and more traditional schemes where reference haplotypes are present in all iterations. One way to do so is to perform cross-validation of the phasing of the study population, creating different subsets where e.g. 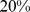 of individuals are left out, counting the number of flips when comparing the resulting haplotypes for individuals common between subsets. Regions of individual genomes where the number of flips are high indicate that the resulting haplotypes are influenced by the information from only a few other individuals in the population, possibly indicating the issue of insufficient chain mixing noted here.

## References

[1] Burton P, Clayton D, Cardon L, Craddock N, Deloukas P, et al (2007) Genome-wide association study of 14,000 cases of seven common diseases and 3,000 shared controls. Nature 447: 661–678. 17554300 10.1038/nature05911 PMC2719288

[2] Turnbull C, Ahmed S, Morrison J, Pernet D, Renwick A, et al (2010) Genome-wide association study identifies five new breast cancer susceptibility loci. Nature Genetics 42: 504–507. 20453838 10.1038/ng.586 PMC3632836

[3] Li Y, Willer C, Sanna S, Abecasis G (2009) Genotype imputation. Annual Review of Genomics and Human Genetics 10: 387–406. 10.1146/annurev.genom.9.081307.164242 PMC2925172 19715440

[4] Druet T, Schrooten C, de Roos A (2010) Imputation of genotypes from different single nucleotide polymorphism panels in dairy cattle. Journal of Dairy Science 93: 5443 –5454. 20965360 10.3168/jds.2010-3255

[5] International HapMap Consortium (2003) The international HapMap project. Nature 426: 789–796. 14685227 10.1038/nature02168

[6] Genomes Project Consortium (2010) A map of human genome variation from population-scale sequencing. Nature 467: 1061–1073. 20981092 10.1038/nature09534 PMC3042601

[7] The 1000 Genomes Project Consortium (2012) An integrated map of genetic variation from 1,092 human genomes. Nature 491: 56–65. 23128226 10.1038/nature11632 PMC3498066

[8] Li Y, Willer CJ, Ding J, Scheet P, Abecasis GR (2010) MaCH: using sequence and genotype data to estimate haplotypes and unobserved genotypes. Genetic Epidemiology 34: 816–834. 21058334 10.1002/gepi.20533 PMC3175618

[9] Howie BN, Donnelly P, Marchini J (2009) A exible and accurate genotype imputation method for the next generation of genome-wide association studies. PLoS Genetics 5: e1000529. 19543373 10.1371/journal.pgen.1000529 PMC2689936

[10] Delaneau O, Coulonges C, Zagury JF (2008) Shape-IT: new rapid and accurate algorithm for haplotype inference. BMC Bioinformatics 9: 540. 19087329 10.1186/1471-2105-9-540 PMC2647951

[11] Abecasis G, Fuchsberger C (2012) Minimac. Available: http://genome.sph.umich.edu/wiki/Minimac.

[12] Scheet P, Stephens M (2006) A fast and exible statistical model for large-scale population genotype data: applications to inferring missing genotypes and haplotypic phase. The American Journal of Human Genetics 78: 629–644. 16532393 10.1086/502802 PMC1424677

[13] Altshuler D, Gibbs R, Peltonen L, Dermitzakis E, Schaffner S, et al (2010) Integrating common and rare genetic variation in diverse human populations. Nature 467: 52–58. 20811451 10.1038/nature09298 PMC3173859

[14] Marchini J, Cutler D, Patterson N, Stephens M, Eskin E, et al (2006) A comparison of phasing algorithms for trios and unrelated individuals. The American Journal of Human Genetics 78: 437–450. 16465620 10.1086/500808 PMC1380287

